# The Lausanne cohort Lc65+: a population-based prospective study of the manifestations, determinants and outcomes of frailty

**DOI:** 10.1186/1471-2318-8-20

**Published:** 2008-08-18

**Authors:** Brigitte Santos-Eggimann, Athanassia Karmaniola, Laurence Seematter-Bagnoud, Jacques Spagnoli, Christophe Büla, Jacques Cornuz, Nicolas Rodondi, Peter Vollenweider, Gérard Waeber, Alain Pécoud

**Affiliations:** 1 Institute of Social and Preventive Medicine, University of Lausanne Hospital Center, 52 route de Berne, 1010 Lausanne, Switzerland; 2 Service of Geriatrics and Geriatric Rehabilitation, University of Lausanne Hospital Center, Lausanne, Switzerland; 3 University of Lausanne Department of Ambulatory Care and Community Medicine, Lausanne, Switzerland; 4 Department of Medicine, University of Lausanne Hospital Center, Lausanne, Switzerland; 5 Department of Community Medicine and Health, University of Lausanne Hospital Center, Switzerland

## Abstract

**Background:**

Frailty is a relatively new geriatric concept referring to an increased vulnerability to stressors. Various definitions have been proposed, as well as a range of multidimensional instruments for its measurement. More recently, a frailty phenotype that predicts a range of adverse outcomes has been described. Understanding frailty is a particular challenge both from a clinical and a public health perspective because it may be a reversible precursor of functional dependence. The Lausanne cohort Lc65+ is a longitudinal study specifically designed to investigate the manifestations of frailty from its first signs in the youngest old, identify medical and psychosocial determinants, and describe its evolution and related outcomes.

**Methods/Design:**

The Lc65+ cohort was launched in 2004 with the random selection of 3054 eligible individuals aged 65 to 70 (birth year 1934–1938) in the non-institutionalized population of Lausanne (Switzerland). The baseline data collection was completed among 1422 participants in 2004–2005 through questionnaires, examination and performance tests. It comprised a wide range of medical and psychosocial dimensions, including a life course history of adverse events. Outcomes measures comprise subjective health, limitations in activities of daily living, mobility impairments, development of medical conditions or chronic health problems, falls, institutionalization, health services utilization, and death. Two additional random samples of 65–70 years old subjects will be surveyed in 2009 (birth year 1939–1943) and in 2014 (birth year 1944–1948).

**Discussion:**

The Lc65+ study focuses on the sequence *"Determinants → Components → Consequences" *of frailty. It currently provides information on health in the youngest old and will allow comparisons to be made between the profiles of aging individuals born before, during and at the end of the Second World War.

## Background

Health and social security systems of industrialized countries are confronted with aging populations and must solve problems related to functional dependence over a wide scale resulting from an epidemic of chronic diseases. This unprecedented situation has prompted researchers to focus their efforts on studying relationships between chronic diseases and the development of disability [1,2], and documenting and forecasting related needs for chronic care. Functional dependency, however, mostly concerns the oldest old population, while demographic trends and population health over the next 30 years will be determined not only by the evolution of longevity, but also by the aging of the large cohort generated by the post-World War II baby-boom. Health and health care needs of this youngest old population have been less well studied. Baby-boomers will be affected by the consequences of cumulated chronic diseases in two decades from now, and preventing disability in this cohort should be considered a public health priority.

A logical approach is to study aging individuals not yet affected by disability. The concept of frailty [3,4] is of particular interest in this regard. A better understanding of the pathway leading from health to frailty and to disability is necessary for preventive intervention. Despite a large volume of recent publications on the subject, and a variety of models, definitions and criteria [5], frailty is still an evolving concept [3,6-8]. There is nevertheless a consensus view that considers frailty as a multidimensional geriatric syndrome with biological, physiological and psychosocial components, and as a state of increasing vulnerability and loss of adaptability to stress [5,9]. Rather than a dichotomous characteristic separating older subjects into two distinct subgroups, it is viewed as a progressive loss of capacity to adapt to complexity and to environmental stressors [10], and as a decline in the ability of an individual to withstand illness without loss of function (functional homeostasis) [11,12]. Campbell and Buchner [13] described frailty as a condition or syndrome which results from a multi-system reduction in reserve capacity to the extent that a number of physiological systems are close to, or past, the threshold of symptomatic clinical failure.

The detection and quantification of frailty in epidemiological studies necessitate some operational definition of this concept. The frailty model proposed by Fried et al. is one of the most frequently used and seems of particular interest for research since it integrates a description of a measurable frailty phenotype within a theoretical concept of causation, manifestations and consequences [14,15]. In this model, the clinical syndrome of frailty is influenced by diseases and by declines in physiologic function and reserve, and it results in adverse outcomes that range from falls to death. The Fried et al. phenotype relies on five items: unintentional weight loss or sarcopenia, weakness as measured by grip strength, poor endurance resulting in self-reported exhaustion, slowness as measured by walking speed, and self-reported low physical activity. It was developed in the context of the longitudinal Cardiovascular Health Study and validated in the Women's Health and Aging Studies [16]. At this stage of knowledge, the phenotype described by Fried et al. seems the most concrete as well as the most agreed upon way to detect frailty. Its frequency has been estimated in a few studies [16-21]. However, despite a consensus on its pertinence, several concerns about this phenotype could be raised. First, this phenotype likely neglects some important dimensions of frailty, as it contains mostly physical characteristics, even though the inclusion of self-reported exhaustion, which is frequently associated with depression, already indicates a contribution of mental health to the frailty syndrome [22]. The Fried phenotype will probably evolve to include additional dimensions such as cognitive and psychological characteristics. Second, the clinical applicability of this phenotype has been questioned and simplified versions need to be developed [23]. Third, there is much debate on the role of psychosocial and economic characteristics in the frailty syndrome. Key components of several multidimensional models of frailty, such as economic vulnerability, may act as determinants, as enhancers, or as outcomes of frailty. Finally, despite a growing body of literature, the chronology and temporal relationships between the different determinants of frailty remain largely speculative.

Improving our knowledge of frailty is particularly appealing because frailty may expose individuals to an increased risk of a range of adverse outcomes and constitute a reversible precursor of functional loss in old age [24,25]. Falls, injuries, acute illnesses, repeated use of emergency services, hospitalizations, disability, and death have been found to be associated with sub-clinical diseases and frailty [15,26-31]. As a result, frailty also appears to be a powerful indicator of health status and of health care needs of aging populations. From a public health perspective, the early detection and prevention of frailty may influence the progression of disability in aging populations [32]. This, however, requires improvements in our understanding of the "Determinants → Components → Consequences" sequence that characterizes age-related frailty.

### Rationale and aims of the Lc65+ study

The rationale for undertaking the Lc65+ study is the paucity of longitudinal epidemiological data specifically collected to improve our understanding of frailty as 1) a phenomenon resulting from various psychosocial and medical influences, 2) a manifestation of abnormal decline in old age, and 3) a cause of evolution towards adverse outcomes, particularly functional decline and a high level of health services utilization. The ultimate goal of the Lc65+ study is to open the field toward developing and testing interventions to potentially reverse the frailty pathway. This study will provide essential information to shape individual and community-based preventive interventions, taking into account the opinions of frail older individuals and their caring environment, and recognizing the evolution of health and expectations across population groups born before, during and after the Second World War.

The specific aims of the Lc65+ cohort are to investigate:

a) the sequence of the physical and mental health manifestations of frailty ***(phenotype)***;

b) the relationship between subjective health and objective manifestations of frailty ***(perception****)*; the extent to which frail individuals perceive their entry and progressions in the spiral of frailty is an essential question in public health, particularly for the quantification of frailty as a major indicator of health in aging populations, since survey data often rely essentially on self-reported data.

c) the trajectories and transitions between levels of frailty ***(natural history)***;

d) the environmental, medical and psychosocial determinants or other predictive factors for frailty ***(risk factors)***;

e) the effect of frailty on the risk of falls, functional impairments or dependency, secondary morbidity, health services utilization and death ***(impact*)**;

f) the self-perceived and objective levels of health and frailty from the age of 65 years in individuals born before, during and after the Second World War ***(public health)***.

## Methods/design

### Design

**Figure 1:**
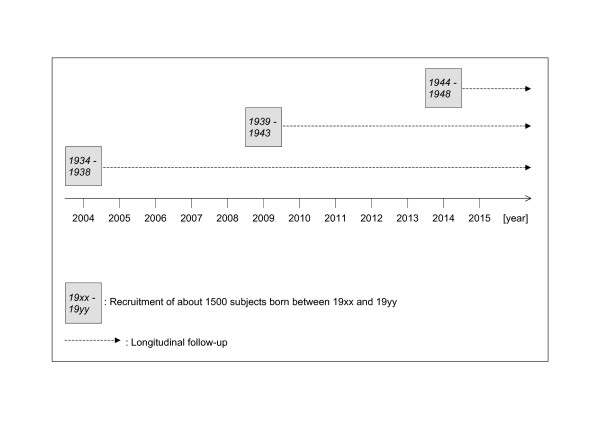
General design of the Lausanne cohort Lc65+ project 2004–2015.

The Lausanne cohort Lc65+ is a longitudinal, observational study initiated and conducted by the Institute of Social and Preventive Medicine at the University of Lausanne Hospital Center (Switzerland), in collaboration with clinical partners from the University of Lausanne Hospital Center (CHUV) and Department of Community Medicine and Health. The study protocol was approved by the Ethics Committee of the Faculty of Biology and Medicine, University of Lausanne. Three successive representative samples of the general community-dwelling population of about 1500 individuals each will be followed from age 65 to death (Figure 1). Subjects are enrolled at the age of 65 to 70 and give written consent for their participation.

### Sampling and recruitment in 2004

**Figure 2:**
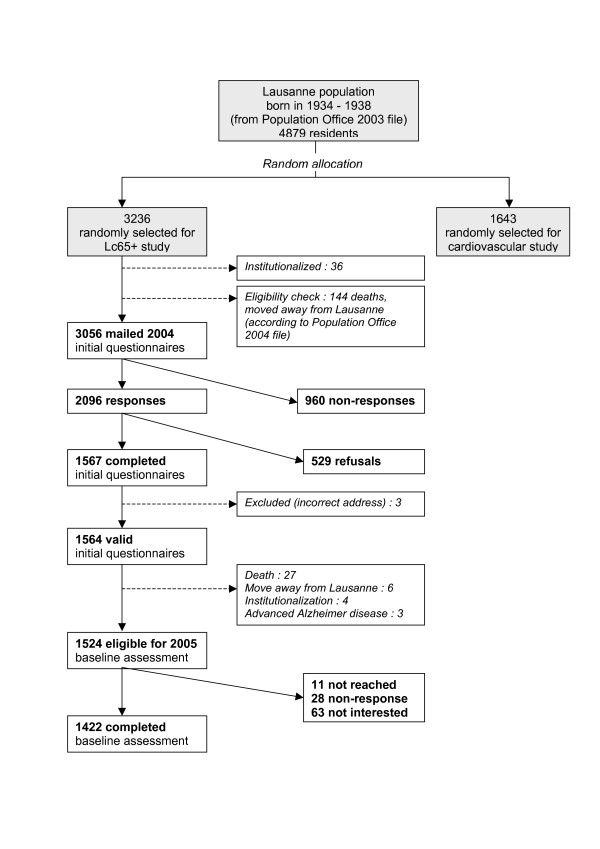
Lausanne cohort Lc65+ Study recruitment flowchart.

The first stage of sampling and recruitment in the Lc65+ study took place in 2004 (Figure 2). A similar procedure will be repeated in 2009 and 2014. Eligibility is defined by the place of residence (Lausanne, a Swiss city of 125000 inhabitants) and by the year of birth. Subjects living in an institution or unable to respond by themselves due to advanced dementia are excluded.

In April 2003, the Population Office extracted a list of city residents comprising 4879 individuals born between 1934 and 1938. All residents in this age category were randomly allocated to two groups for participation either in a study of cardiovascular diseases (N1 = 1643, 33.7%) or in the Lausanne cohort Lc65+ study (N2 = 3236, 66.3%), which resulted in a selection by simple random sampling for each of these two studies. Of the 3236 Lausanne residents randomly allocated to the Lc65+ study, 36 (1.1%) individuals living in an institution were excluded, 144 (4.5%) persons were further excluded on the basis of an updated list issued by the Population Office in 2004 (dead or moved away from Lausanne) and 3056 residents were considered eligible for contact by mail.

In March 2004, all selected individuals received a support letter from the Surgeon General of the Canton of Vaud, followed one week later by a mailing including a presentation of the study, an initial self-administered questionnaire and a stamped return envelope. Non-respondents received two follow-up mailshots with the same contents. The last mailing included an anonymous form for reporting refusals and corresponding reasons.

Out of the 3056 mailed questionnaires, 2096 (68.6%) responses were registered; 1567 (74.8%) persons agreed to participate and 529 (25.2%) refused. Compared to non-respondents or refusers, participants did not differ in gender (41.3% men in participants versus 41.4% in non-participants, χ^2 ^test p = 0.9) or in birth year distribution (in men: 1934 18.1% versus 18.6%, 1935 22.3% versus 19.8%, 1936 20.2% versus 22.5%, 1937 19.9% versus 16.5%, 1938 19.5% versus 22.5%, χ^2 ^test p = 0.3/in women: 1934 21.1% versus 19.9%, 1935 20.3% versus 20.1%, 1936 20.4% versus 18.8%, 1937 17.9% versus 20.8%, 1938 20.2% versus 20.4%, χ^2 ^test p = 0.6). Participants' socio-economic characteristics closely reflected the Lausanne general population in the same age category in aggregate statistics from the Population Office (proportions of foreign nationality, distribution of marital status) or from the 2000 Swiss national population census (nationality, marital status, place of birth, living arrangement, professional activity – data not shown). Refusals were mostly motivated (multiple reasons possible) by a general disinclination to participate in any survey (57.8%), or to agree to follow-up contacts (53.9%); 24% of refusers considered that some questions intruded on their privacy, 17.8% did not have the time or lacked interest in the study topic, 17.0% refused to participate in a non-anonymous data collection. Some 10.6% indicated language limitations, 7.8% expressed difficulty in understanding questions and the same proportion attributed their refusal to poor health.

Of the 1567 respondents to the initial questionnaire, 3 subjects were later considered as ineligible (incorrect address in 2004), leaving 1564 valid observations. In 2005, all participants were invited to complete the baseline survey; 1524 (97.4%) were still eligible; 1422 (93.3%) participated in the assessment and 1416 could be classified as non-frail, pre-frail or frail according to the Fried et al. phenotype [15].

An additional sample of 100 residents born in 1933 was selected in 2004, following the same rules and process, for the piloting of questionnaires as well as in-person interviews and performance tests conducted by medical research assistants.

### Baseline assessment in 2004–2005

Baseline data are collected using a two-steps procedure involving a self-administered mailed questionnaire at recruitment, followed by an in-person interview at the study center with anthropometric measurements and performance tests performed by trained medical assistants. Table 1 summarizes the contents of the Lc65+ baseline assessment.

**Table 1:** Contents of Lausanne cohort Lc65+ Study 2004–2005 baseline data collection.

**Self-completed questionnaire**
- Childhood history: premature birth and birth weight category, family size at birth and at the age of 10, economic environment at birth and change in childhood, major diseases and injuries, stressful life events during infancy and early adolescence
- Socio-economics: country of birth, nationalities, achieved education, type and duration of professional activity, current working activity and circumstances of retirement; current subsidized health insurance as an indicator of low income, stressful life events in adulthood, marital status, number of children, size and composition of household
- Subjective health (WHO formulation) absolute and relative to contemporaries; perception of own aging; fear of disease, weakness, sleep perturbation, according to questions extracted from Swiss Health Surveys; sight and hearing impairments; medical diagnoses, chronic symptoms
- Screen for mental health and depression (GHQ-12) [33,34]
- Health-related behaviors: current physical activity, decrease in physical activity in past twelve months, smoking history, alcohol consumption (WHO Audit-C) [35,36]
- Screen for difficulty and dependence in basic and instrumental activities of daily living
- Current height and weight, weight 5 years ago, unintentional weight loss
- Falls, fear of falling and impact on activities, falls efficacy (FES-I) [37]
- Stressful life events in past 12 months (GALES Part I: list of events) [38]
**Interview**
- Stressful life events in past 12 months (GALES Part II: level of stress and feelings) [38]
- Nutrition (MNA [39-41], completed by questions on nutritional habits developed in the Canadian NuAge project [42])
- Health services utilization in past twelve months (as assessed in SHARE) [43]
- Self-assessment of the economic situation
**Measurements**
- Weight and height
- Arm, waist, hip, and calf circumferences; biceps, triceps and supra-iliac skinfolds (GPM^® ^caliper)
- Resting blood pressure and heart rate (measured three times at 5–10 minute intervals on right arm, OMRON^® ^digital automatic blood pressure monitor, manually in case of rhythm abnormalities)
**Performance tests**
- Grip strength test on the right hand (Baseline^® ^hydraulic dynamometer three measurements) [44-46]
- Moberg Picking-Up Test on dominant hand [47]
- Balance tests (10 seconds side-by-side, semi-tandem and tandem standing with open eyes according to the protocol of EPESE, 1 minute side-by-side standing, open and closed eyes) [48]
- Timed Up-and-Go test [49-51]
- Self-selected walking speed (20 meters walk single task, double task: walk and backward count, double task: walk and water glass, triple task: walk, backward count and water glass) [52-54]
- Timed five chair rises
- Cognition test (MMSE) [55], frontal and temporo-parietal functioning (Clock Drawing Test) [56-58]. If MMSE ≥ 24: verbal fluency (fruit and vegetables in one minute) [59], Trail Making Test parts A and B [60,61]

#### Initial questionnaire (2004)

The initial questionnaire has been designed to enable comparisons to be made with other major population-based health surveys conducted in Switzerland and Europe. Questions included batteries already used in the Swiss health surveys (Federal Office for Statistics), in the MONICA study [62] or in the SHARE European survey [43]. The instrument was pre-tested first on a convenience sample of 9 volunteers and then on 42 randomly selected subjects born in 1933. Contents emphasized life history, with indications of socio-economic status and main medical diagnoses in childhood and adulthood, and current health. As events from the past are liable to be remembered imperfectly [63], the questionnaire was organized in chronological sections from childhood to current health status in order to enhance recall.

#### Completion of baseline data collection (2005)

The 2005 assessment was performed according to a standardized protocol by medical research assistants supervised by a senior psychologist, after two weeks of specific training at the study center followed by a pre-test on the pilot random sample of subjects born in 1933. A self-administered questionnaire was sent to the subjects' homes prior to the appointment and responses were checked for coherence and completeness by the medical assistants. Dimensions, instruments and tests included in interviews and examinations are detailed in Table 1. Finally, participants were asked to sign informed consent forms for continuing follow-up and for linking data collected in the Lc65+ with death and hospital discharge statistics.

#### Frailty assessment

Frailty was assessed at baseline according to the five characteristics (shrinking, weakness, exhaustion, slowness and low activity) included in the frailty phenotype described by L. Fried et al.; Table 2 summarizes how each characteristic was operationalized in the Cardiovascular Health Study [15] and in the Lc65+ study.

**Table 2:** Operationalization of frailty characteristics in the Cardiovascular Health Study (CHS) [15] and in the Lausanne cohort Lc65+ Study.

	**Criteria**	
	**Cardiovascular Health Study**	**Lausanne cohort Lc65+ Study**
**Characteristic of frailty**		
Shrinking	Unintentional weight loss >10 lbs in prior year	Any reported unintentional weight loss in prior year
Weakness	Grip strength: lowest 20% by gender and body mass index	Grip strength: application of CHS gender and body mass index specific cut-off values
Poor endurance, exhaustion	Exhaustion self-report: responds *a moderate amount of the time *or *most of the time *to either statement "I felt everything I did was an effort" or "I could not get going" in the last week	Exhaustion self-report: responds *much *to "Did you have feelings of generalized weakness, weariness, lack of energy in the last four weeks?"
Slowness	Walking time/15 feet: slowest 20% by gender and height	Walking time/20 meters: application of CHS gender and height specific cut-off values
Low activity	Physical activity self-report: lowest 20% Kcals/week expenditure, by gender, estimated from the short version of the Minnesota Leisure Time Activity questionnaire	Physical activity self-report: less than 20 minutes of sport activity once a week and less than 30 cumulated minutes walk per day 3 times a week and avoidance of stairs climbing or light loads carrying in daily activities

**Classification of frailty**		
Non-frail or robust	0 criterion present	0 criterion present
Intermediate, possibly pre-frail	1–2 criteria present	1–2 criteria present
Frail	3–5 criteria present	3–5 criteria present

### Follow-up

The Lc65+ follow-up includes an annual self-administered questionnaire (or an interview questionnaire in case of deteriorated health or cultural circumstances). Mailed questionnaires also apply to individuals who moved away from the study area, where these can be located. In addition, subjects are submitted every third year to an interview and an examination performed at the study center, replicating physical and mental performance tests already included in the baseline data collection. This follow-up process monitors all subjects until death, refusal, loss to follow-up, long-term residence in a nursing home of subjects with cognitive impairment that precludes them from responding, or hospice care. Specific problems such as impaired vision or home confinement are resolved by adapting the data collection process (e.g. phone interviews rather than mailed questionnaire, home visit rather than appointment at the study center). Furthermore, with the written consent of participants, a passive follow-up will be organized (file linkage with death certificates, possibly with hospital discharge records if feasible) until death or refusal. At all steps of recruitment and follow-up, non-responders are re-contacted by various ways (phone, mail). Where necessary, details of two relatives or friends obtained on recruitment in order to facilitate follow-up contacts can be used to reach the cohort members. Inactive addresses are checked with the Population Office.

Of 1422 participants enrolled in the Lc5+ study in 2005, 1344 (94.5%) returned completed questionnaires in 2006, 18 had died, entered institutions with impaired cognitive functions, moved away permanently or were away from Lausanne for a prolonged period; 2 subjects could not be found in spite of a valid address, 17 could not participate this year but did not retire from the cohort, and 41 asked to quit the study. In 2007, 1309 (92.1% of 2005 participants) returned their completed questionnaire; 19 had died, 17 had moved away from Lausanne and 5 had entered an institution with cognitive problems.

#### Outcomes

The annual follow-up basically purports to study outcomes such as self-rated health, morbidity, reduced activity, functional decline in instrumental and basic activities of daily living, health services utilization and death. In addition, interviews and examinations performed every third year are designed to study the health-related quality of life, objective changes in physical and mental health performance, as well as changes in dimensions of the frailty phenotype.

The 2006 and 2007 self-administered follow-up questionnaires covered:

- subjective health, fear of disease, weakness, sleep perturbation, screen for depression;

- medical diagnoses and treatments in past 12 months;

- chronic disturbing signs and symptoms lasting more than 6 months;

- current drugs;

- stressful life events in the past twelve months;

- unintentional weight loss, falls, fear of falling in the past 12 months;

- physical activity, changes in physical activity in the past 12 months;

- current difficulties/impairments in mobility tasks;

- current difficulties or help received for health-related reasons in Katz' BADLs and in Lawton IADLs;

- pain limiting activities in the past 4 weeks;

- medical visits, emergency room consultations, hospitalizations, home care and help in the past 12 months;

- current paid and unpaid work.

Yearly follow-up questionnaires also enable additional dimensions to be investigated or selected dimensions to be explored in more depth. The 2006 questionnaire integrated an assessment of the social network (abbreviated version of LSNS II [64,65]; items from the MOS Social Support Survey [66]). In 2007, participants in the Lc65+ study were asked to fill out a complementary questionnaire on sexuality in order to explore relationships with health; owing to the sensitive nature of this domain, this questionnaire was presented as optional.

In 2008, the first triennial follow-up interview and examination of the data collection in progress covers the same contents as the 2005 baseline, with some elements added from the annual self-administered questionnaires (e.g. detailed information on mobility and ADL difficulties). An assessment of health-related quality of life based on a standardized instrument (MOS SF-12) was also added, while information collected on nutritional habits and on stressful life events have been slightly simplified.

### Data check and analyses

All questionnaires, interview and examination forms are first checked by a trained researcher. The quality of data entry is systematically verified to detect errors. Analyses will combine retrospective (e.g. for the study of early life experiences as risk factors for frailty), cross-sectional (e.g. for the study of relationships between contemporaneous measurements of a frailty phenotype and mental performance included in baseline data collection) and prospective (for a majority of research questions, e.g. concerning the predictors of frailty or the outcomes of frailty) approaches. The variety of dimensions included in the Lc65+ study will enable us to control for a wide range of factors in analyses or multivariate models.

At baseline, in the Lc65+ study, the estimated proportions for non-frail, intermediate (possibly pre-frail) and frail subjects were 71.1%, 26.4%, and 2.5%, respectively, in 1283 subjects with complete information on all five dimensions in the frailty phenotype defined by L. Fried et al. Applying rules used in the Cardiovascular Health Study, in which subjects considered as evaluable for frailty had three or more non-missing frailty components among the five criteria [15], 1416 subjects were classified as non-frail (71.6%), intermediate, possibly pre-frail (26.3%) or frail (2.3%).

## Discussion

In the past 50 years, persons aged 80+ have been the fastest growing segment of the population in Switzerland. The current very old population was born before 1928 and its growth has hitherto essentially been due to gains in life expectancy observed throughout the 20th century. We already face difficulties in organizing and financing the resource-intensive care associated with this age. According to conservative demographic projections, the number of Swiss residents aged 80+ will peak in 2050 [66]. This trend is common to most industrialized countries. Understanding the frailty process and specific health characteristics of cohorts born just before, during and after the Second World War is crucial to prevent their evolution towards increasing frailty and disability. Most evaluations of preventive actions (e.g. home visits) pointed to a greater effectiveness in less dependent subjects [68-70], suggesting that interventions in pre-dependent, frail individuals is probably an appropriate strategy.

To our knowledge, the Lc65+ is the first cohort specifically designed to study the frailty process in the general population with an emphasis on the youngest old. The low proportion of frail individuals at recruitment confirms the potential of this cohort for studying the occurrence and the evolution of frailty from its initial manifestations. Consequently, it will provide innovative longitudinal data on which to build the multidisciplinary research required to elaborate preventive interventions targeting frail individuals. A prospective design is necessary to disentangle the respective contributions of all medical and psychosocial characteristics encompassed within the frailty concept, study the temporal sequence of mental and physical loss of homeostasis in the frailty process, and distinguish elements that act as risk factors, determinants and facilitators in order to define appropriate interventions. A cohort design is also the only method providing accurate information concerning the impact of frailty on later outcomes such as the development of functional dependence.

The strong methodological design, the inclusion of a broad range of dimensions and risk factors, the successful enrollment – and, so far, retention strategies – are strengths of the Lc65+ project, which will make a substantial contribution towards clarifying the causal pathways leading from health to frailty and to disability.

## List of abbreviations used

ADLs: Activities of daily living; BADLs: Basic activities of daily living; EPESE: Established populations for epidemiologic studies of the elderly; FES-I: Falls efficacy scale -International; GALES: Geriatric adverse life events scale; GHQ-12: General health questionnaire-12; IADLs: Instrumental activities of daily living; LSNS II: Lubben social network scale II; MNA: Mini nutritional assessment; MONICA: Monitoring of trends in cardiovascular diseases; NuAge: Study on nutrition as a determinant of successful aging; MMSE: Mini-mental state examination; MOS: Medical outcome study; MOS SF-12: Medical outcome study – Short form 12; SHARE: Survey of health, aging and retirement in Europe; WHO Audit-C: World health organization Alcohol use disorders identification test – C.

## Competing interests

The authors declare that they have no competing interests.

## Authors' contributions

BSE, principal investigator, drafted the manuscript, initiated the Lc65+ study and is responsible for its design, conduct and analysis. AK, psychologist, participates in the selection of study instruments in the domain of mental health and life events and is responsible for the data collection and the supervision of medical assistants. LSB, physician, participates in the supervision of medical assistants and in data analyses. JS, statistician, is in charge of the Lc65+ study database, conducts and supports data analyses. As members of the Lc65+ study committee, CB, JC, AP, NR, PV and GW are involved in the development of the study, obtaining research funding and selecting instruments. All authors participated in the critical revision of this manuscript.

## Pre-publication history

The pre-publication history for this paper can be accessed here:


